# A Novel Panmacular Strategy for Subthreshold Micropulse Laser in CSC: Two-Year Functional and Morphological Outcomes

**DOI:** 10.3390/jcm15010043

**Published:** 2025-12-20

**Authors:** Małgorzata Latalska, Sławomir Dresler, Magdalena Wójciak, Ireneusz Sowa, Robert Rejdak

**Affiliations:** 1 Chair and Department of General and Pediatric Ophthalmology, Medical University of Lublin, 20-079 Lublin, Poland; robert.rejdak@umlub.edu.pl; 2 Department of Analytical Chemistry, Medical University of Lublin, Chodźki 4a, 20-093 Lublin, Poland; slawomir.dresler@umlub.edu.pl (S.D.); magdalena.wojciak@umlub.edu.pl (M.W.); ireneuszsowa@umlub.edu.pl (I.S.); 3 Department of Plant Physiology and Biophysics, Institute of Biological Sciences, Maria Curie-Skłodowska University, Akademicka 19, 20-033 Lublin, Poland

**Keywords:** panmacular treatment, micropulse laser, retinal sensitivity, central serous chorioretinopathy, choroidal thickness, pachyvessel, subretinal fluid, vessel density

## Abstract

**Background/Objectives**: Central serous chorioretinopathy (CSC) is the fourth most common retinal disorder. Subthreshold micropulse laser therapy (MPLT) is a promising option. The study aimed to evaluate whether a novel panmacular MPLT strategy leads to significant long-term functional and structural improvement in acute (aCSC), chronic (cCSC), and recurrent (rCSC). **Methods**: This retrospective, non-comparative study included CSC patients (n = 41) and healthy controls (n = 14). Participants underwent panmacular subthreshold MPLT. Best-corrected visual acuity (BCVA), retinal sensitivity (RetSens), central choroidal thickness (CCT), and pachyvessel diameter were measured at baseline and at 24 months post first treatment. The mean post-intervention observation time (from the last treatment to the final follow-up) was 24.0 months for the aCSC group and 18.6 months for the cCSC and rCSC groups. Treatment required a mean of 1 session in the aCSC group (range 1–3) and a mean of 2.8 sessions in the cCSC and rCSC groups (range 1–8). **Results**: Complete subretinal fluid (SRF) resolution was achieved in 100% of aCSC cases, 86.66% of cCSC, and 63.63% of rCSC. Non-resolution/recurrence occurred in 13.33% of cCSC (2/15), and 36.37% of rCSC (4/11). After two years BCVA significantly improved across all groups (e.g., aCSC: improved from 0.74 ± 0.05 to 0.98 ± 0.02, *p* = 0.005). RetSens significantly improved in aCSC (increased by 5 dB, *p* = 0.001) and cCSC (increased by 3.8 dB, *p* = 0.046) CCT, CRT and pachyvessel diameter significantly decreased post-treatment in all CSC groups (*p* < 0.05). **Conclusions**: Panmacular MPLT is a safe and effective therapy for CSC, providing significant long-term functional and structural improvement, particularly in acute and chronic cases. The therapeutic effects were weakest in the rCSC group, which showed the poorest functional outcomes and a higher burden, suggesting that a recurrent disease course may be a primary negative prognostic factor.

## 1. Introduction

Central serous chorioretinopathy (CSC) is the fourth most common retinal disease, precede by age-related macular degeneration (AMD), diabetic retinopathy (DR), and central retinal vein occlusion (CRVO). It primarily affects men of working age (male-to-female ratio, 3:1) and is currently classified as a disease within the pachychoroidopathy spectrum [1]. Its primary feature is thickening and pathological hyperpermeability of the choroid, which leads to secondary dysfunction of the retinal pigment epithelium (RPE) and accumulation of subretinal fluid (SRF) [1].

Although the primary cause of CSC remains unknown since its first description by von Graefe in 1866 [2], a growing body of evidence points to venous stasis in the choroid as a key element in its pathogenesis [3,4,5,6]. It is believed that congestion in Haller’s layer is the result of impaired venous outflow, leading to the formation of pachyvessels and pachychoroid [7]. There may be two causes for this stasis. Firstly, vascular anomalies such as dilation and asymmetry of the vortex veins, which are observed in over 80% of CSC cases and the presence of abnormal inter-vortex venous anastomoses that disrupt normal blood flow have been identified [4,6,8]. Secondly, the existence of a mechanical blockage of venous outflow is suggested and supported by the results of recent studies, which have shown increased scleral thickness in eyes with CSC [6,7,8]. The thickened sclera may exert external pressure on the choroidal veins, further exacerbating blood stasis in the choroid [6,7,8]. Significant visual impairment (defined as BCVA < 0.5) may occur in approximately 13% of patients. Recurrences occur in 15–50% of patients, and progression from acute to chronic CSC occurs in approximately 16% of patients [1]. Therefore, prevention and elimination of multiple risk factors, along with appropriate treatment, are crucial to preserving vision in this group of patients.

The complex interaction between the retinal and choroidal microvasculature is fundamental to maintaining normal visual function. Impairment in either system, caused by systemic or localized disease, can lead to significant structural and functional damage. One example of this is CSC, which involves abnormal thickening and hyperpermeability of choroidal vessels. This condition ultimately leads to secondary dysfunction of the retinal pigment epithelium (RPE) and accumulation of subretinal fluid (SRF).

The chronic choroidal overload causes secondary damage to the overlaying RPE and choriocapillaris, resulting in atrophy and localized vascular compromise [1]. The loss of RPE integrity and the persistent accumulation of SRF directly impair the function of photoreceptors and Müller cells [9]. Studies have confirmed the destructive effects of short-term SRF on retinal function and structure [10,11,12].

This RPE dysfuntion resembles microcirculatory changes observed in other retinal disease, such as diabetic macular edoema (DME) [9,13,14]. In DME MPLT treatment can be effective by stimulating RPE and Müller cells and modulating retinal neuroinflammatory biomarkers [9,13,15]. The structural integrity of the inner retina, particularly the Ganglion Cell Complex (GCC) thickness, constitutes a critical prognostic parameter influencing the ultimate functional outcome following any intervention for Central Serous Chorioretinopathy (CSC).

The underlying vascular predisposition in CSC is often linked to systemic factors like stress, steroid use, and smoking. Systemic microvascular changes in CSC patients have been observed using nailfold videocapillaroscopy (NVC), widely used to assess microcirculation, for example, in rheumatoid systemic disorders [16,17,18]. The observed correlations between distant capillary beds and ocular vascular parameters suggest a broader, systemic microvascular dysfunction underlying CSC.

The panmacular MPLT protocol is founded on the principle of maximizing the cellular stimulation area to enhance therapeutic effects, known as the “mass effect” [13,19]. Since MPLT stimulates RPE and Müller cells to restore function, expanding the treatment area beyond the site of neurosensory detachment to the entire macula is hypothesized to provide a superior therapeutic response, promoting healing across the affected RPE layer [9,13]. This approach aims to address RPE dysfunction over a wider region, accelerating SRF resolution (H1: Therapeutic effect hypothesis), normalizing retinal structure and function (H2: Normalization hypothesis), and potentially promoting beneficial changes in choroidal vascular hemodynamics, as suggested by the monitored reduction in CCT and pachyvessel diameter.

Determining the optimal treatment for CSC and its optimal timing remains an ongoing challenge. Typically, acute CSC is not treated due to its self-limiting nature. Many inconsistencies arise in the management of recurrent and chronic CSC. Laser treatment has been generally avoided because of the apprehension of side effects typical of conventional laser therapy. The exception is photodynamic therapy (PDT) for chronic CSC, which showed superior results to other treatments, such as MPLT, in the large PLACE trial [20]. However, it should be noted that according to the MPLT methodology used in this study, the laser was limited only to the area of the neurosensory retinal detachment, mirroring PDT. Despite this similarity, these two therapies have different modes of action. Numerous studies have demonstrated that subthreshold MPLT can effectively stimulate RPE and Müller cells, improving the transretinal pump and eliminating subretinal fluid [9,13,15]. Therefore, it seems clear that maximizing the stimulation area can enhance therapeutic effects [13]. Thus, the principle of the panmacular MPLT protocol is based on the extension concept of obtaining a “mass effect” of cellular stimulation (within the major vascular arcades on the whole macular area) [13,19,21].

There is currently a need to reach a consensus on how to define and determine the duration of different types of CSC. Central Serous Chorioretinopathy International Group proposed a new classification for CSC [22]. However, the proposed timeframe to distinguish between simple and complex CSCs is still six months, which is questionable because the risk of RPE damage and permanent visual impairment is too high. Studies have confirmed the destructive effect of short-term subretinal fluid in patients with resolved acute CSC [11].

This lack of agreement leads to difficulties in establishing a specific timeframe for treatment, ultimately hindering the prevention of the negative consequences of chronic CSC. Additionally, a standardized treatment protocol for Micropulse Laser Therapy (MPLT) in CSC should be used to compare treatment outcomes with other trials in the future. While numerous reports in the literature have evaluated the efficacy of various treatment modalities for CSC, MPLT has often been confined to treating the area of neurosensory retinal detachment, prioritizing foveal preservation whereas in DME, laser treatment of the entire area of edoema and even the inter-vascular zone is recommended [10,14,20,23,24]. Recently, transfoveal MPLT was introduced as a treatment for acute CSC, but different results were obtained [25,26].

The aims of the study were to: (1) assess whether a new protocol of MPLT—panmacular therapy leads to statistically significant improvement in functional and structural retinal parameters within each CSC group; (2) determine whether, following therapy, the parameters of treated CSC eyes approach those observed in healthy controls. To achieve these aims, the following hypotheses were tested: Therapeutic effect hypothesis (H1): Panmacular MPLT statistically significant improvement in functional and morphological parameters within each CSC group during the two-year follow-up. Normalization hypothesis (H2): After panmacular MPLT, the functional and morphological parameters of treated CSC eyes approach or reach levels comparable to those of healthy controls.

## 2. Materials and Methods

### 2.1. The Study Groups

This study is based on 41 eyes of 41 patients treated with panmacular MPLT, selected from a cohort of 152 patients diagnosed with CSC at the Ophthalmology Department of the Medical University of Lublin between December 2018 and December 2023, who underwent panmacular subthreshold micropulse 577 nm laser treatment (IQ 577; IRIDEX, Mountain View, CA, USA). The final study group size (n = 41) resulted primarily from the rigorous inclusion/exclusion criteria (e.g., excluding patients with high myopia or severe systemic diseases) and, most importantly, the fact that only these patients completed the full, two-year follow-up protocol with all required multimodal imaging examinations. Based on clinical evaluation and multimodal imaging findings, patients were classified into acute (aCSC), recurrent (rCSC), and chronic (cCSC) groups (mean age: aCSC 40.9 ± 5.63, rCSC 44 ± 8.96, and cCSC 46.8 ± 6.58). Furthermore, 14 healthy subjects were selected from 41 healthy individuals matched by gender and age who underwent all the analyzed examinations after two years. All participants provided written informed consent before the procedure. The study adhered to the principles of the Declaration of Helsinki, and the design was approved by the local ethical committee at the Medical University of Lublin (KE-0254/291/2018, 29 November 2018). This study was conducted among pre-qualified patients for the nailfold videocapillaroscopy study, as outlined in the previous reports [17,18]. The inclusion and exclusion criteria for the pre-qualified nailfold videocapillaroscopy study were identical to those detailed below for the CSC patient and healthy control groups.

**Figure 1:**
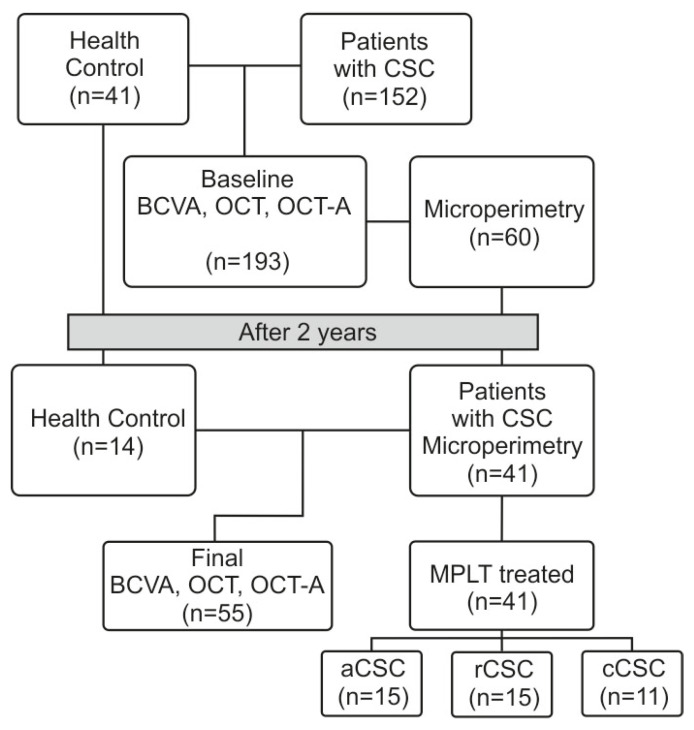
Study design flow chart. From an initial pool of 193 subjects (152 CSC patients and 41 healthy controls), 55 subjects completed the 2-year follow-up with complete data (14 controls, 41 CSC patients) The CSC patient group (n = 41) was divided into aCSC (n = 15), cCSC (n = 15), and rCSC (n = 11). CSC—central serous chorioretinopathy, OCT—optical coherence tomography, BCVA—best corrected visual acuity, OCT-A—optical coherence tomography-angiography, MPLT—micropulse laser treatment, aCSC—acuteCSC, rCSC—recurrent CSC, cCSC—chronicCSC.

The study design flow chart is presented in Figure 1.

The inclusion criteria for the CSC group were the presence of an active CSC in one eye, identified as a serous detachment of the neuroretina. This was confirmed through clinical and OCT examination. The fellow non-active eye was also evaluated.

Patients diagnosed with active CSC were categorized into subgroups utilizing criteria established by the Central Serous Chorioretinopathy International Group [9]. However, we modified the criteria by reducing the observation period from 6 to 3 months. We reduced the time to three months, based on robust evidence that even short-term SRF can be destructive to avoid damage to the RPE layer and photoreceptors [10,11,12]. This modification was made to aggressively prevent the high risk of RPE damage and permanent visual impairment associated with prolonged SRF presence, which is a concern even with the current 6-month recommendation. Treatment was started when SRF did not resolve, remained stable or increased within the first three months from the beginning of disease. In such cases, delaying treatment further, as per some protocols (e.g., 6 months) was deemed to pose a significant risk of permanent damage to the RPE.

1. Acute CSC (aCSC): First known episode of SRF with a total area of RPE alteration ≤ 2 DA (disk area) and/or persistent SRF for less than 3 months.
2. Recurrent CSC (rCSC): Presence of SRF with history or sign of resolved episode with a total area of RPE alteration ≤ 2 DA and/or persistent SRF for less than 3 months.
3. Chronic CSC (cCSC): Presence of persistent SRF > 3 months, with outer retina atrophy including ONL thinning and/or ELM disruption and/or EZ attenuation and total area of RPE alteration > 2 DA or multifocal (including gravitational tract).

The exclusion criteria from the CSC group were as follows: 1. General vascular and neoplastic disorders (e.g., peripheral artery disease, abdominal aortic aneurysm, carotid artery disease, pulmonary embolism, chronic venous insufficiency, anemia). 2. History of anti-cancer treatment. 3. Debilitating conditions such as chronic alcoholism or drug addiction. 4. Concurrent ocular and retinal disease affecting visual acuity, including diabetic retinopathy, age-related macular degeneration, vitreomacular disorders, presence of profound RPE atrophy in the fovea and cystic degeneration in the macula on structural OCT. 5. Myopia exceeding six diopters due to its significant impact on choroid thickness.

The health control group was examined to exclude any ocular diseases. That group included 6 female and 8 male (mean age 45 ± 9.26) healthy volunteers.

### 2.2. Ophtalmologic Examination

All participants, including patients with CSC and healthy individuals, underwent an examination to assess their best corrected visual acuity (BCVA) and fundus ophthalmoscopy at the beginning of the study. Ultra-widefield color and autofluorescence fundus photography were taken using Optos California (Optos, Inc., Marlborough, MA, USA). Visual acuity was assessed using Snellen charts, which were placed at a distance of 5 m in a room with standardized illumination. BCVA was measured with the best correction obtained through subjective refraction.

Spectral domain OCT and OCT-angiography (OCT-A) were conducted using Angio Retina QuickVue, Angio Retina, and Cross Line scans (Algorithm Version A2017,1,0,151, Optovue, Inc., Fremont, CA, USA) following pupil dilation. The scans with the highest resolution were acquired in the central 3 × 3 and 6 × 6 mm^2^ areas, centered on the foveola. Qualitative features and quantitative measures of central retinal thickness (CRT), subfoveal subretinal fluid height (SRF), pigment epithelium detachment (PED), ellipsoid zone disruptions (EZ), alteration in retinal pigment epithelium (RPE), such as disruption or atrophy, and the presence of intraretinal hyperreflective foci (HF) were reviewed and analyzed in the OCT images of all subjects.

Central retinal thickness (CRT) was automatically measured in the central 1.0 mm circle of the EDTRS grid from the inner limiting membrane (ILM) to the outer boundary of the RPE, following a uniform method used in previous studies for result comparison.

SRF height was measured manually in the fovea as the distance between the top of the SRF and the RPE-Bruch’s membrane complex.

**Figure 2:**
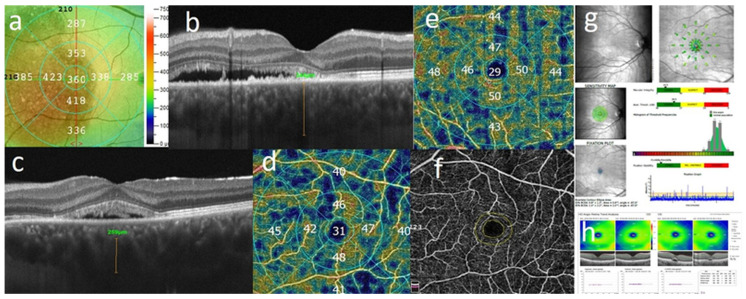
Multimodal imaging features: (**a**) CRT (Central Retinal Thickness), (**b**) Pachyvessel CCT (Central Choroidal Thickness), (**c**) Diameter of pachyvessel, (**d**) Vessel Density of Superficial Plexus, (**e**) Vessel Density of Deep Plexus, (**f**) FAZ (Foveal Avascular Zone), (**g**) microperimetry, (**h**) GCC analysis.

Central choroid thickness (CCT) was manually measured from the outer hyperreflective line corresponding to the RPE-Bruch’s membrane layer to the inner hyperreflective surface of the sclera under the foveal center (Figure 2a). The diameter of the largest visible hyperreflective lumen of the choroid venous (pachyvessel) found on a horizontal Cross-link scan was measured, following the method described by Yang et al. [27] (Figure 2b). These pachyvessels, as per Spaide et al., are formed by the intervortex venous anastomoses primarily located in the central macula in eyes with CSC [3]. Measurements were performed twice, 7 days apart. Cohen’s kappa coefficient was used to assess agreement between the first and second assessments. Measurements with a k coefficient > 0.8 were included in the analysis.

The superficial capillary plexus (SVD) was automatically detected between the internal limiting membrane (ILM) and the inner plexiform layer (IPL). In contrast, the deep capillary plexus (DVD) was identified between the IPL and the outer plexiform layer (OPL). Superficial and deep vessel density (VD) were measured automatically using the AngioAnalytics software on the OptoVue system (Version 2017.1.0,151) (Figure 2c,d). Vessel density (VD) was defined as the proportion of the vessel area with blood flow over the total measured area. The foveal avascular zone (FAZ) was evaluated using the software provided in the OptoVue system (Figure 2e). FAZ was defined as a region within the fovea centralis at the retina’s center devoid of retinal blood vessels.

Mean Retinal Sensitivity (RetSens) was measured by microperimetry using Macular Integrity Assessment microperimeter MAIA II and analyzed as an Average Threshold by analysis software (MAIA II; 2009, CenterVue, Padova, Italy) (Figure 2f). GCC thickness was calculated automatically in superior, inferior hemi-sphere (SupHem and InfHem) and ETDRS Grid by software built in OptoVue system (Figure 2g).

Researchers evaluated well-established risk factors associated with CSC, such as smoking, stress, steroid use, xylometazoline, phosphodiesterase inhibitor intake, diabetes mellitus, autoimmune diseases, insomnia, and obstructive sleep apnea, through a comprehensive analysis of relevant reports.

### 2.3. 577 nm Panmacular Micropulse Laser Treatment

The surgeon performed the treatment in cases where SRF persisted, or its height remained unchanged or increased after three months, MPLT utilizing a 577 nm laser (IQ 577; IRIDEX, USA) was performed by the same experienced surgeon on the entire macula within the major vascular arcades (panmacular protocol), including the fovea. This panmacular strategy is innovative in CSC treatment as it expands the traditional treatment area (usually confined to the area of neurosensory retinal detachment) to the entire macular region, leveraging the “mass effect” principle of cellular stimulation to maximize therapeutic effects on the RPE and Müller cells. This protocol of MPLT strategy has been used in DME treatment [13]. The laser foci used had a diameter of 200 µm and were applied in a confluent manner, ensuring uniform coverage of the entire macular area, from fovea to the major vascular arcades. Consistent laser parameters were used in all eyes, with a duty cycle of 5%, 300 mW, and a duration of 200 ms. BCVA, GCC, and RetSens were assessed at baseline and two-year follow-up visits. The mean post-intervention observation time (from the last treatment to the final follow-up) was 24.0 months for the aCSC group and 18.6 months for the cCSC and rCSC groups. Additionally, CCT, pachyvessel diameter, FAZ, and measurements of the superficial and deep vascular retinal plexus were compared between CSC patients and healthy individuals. Fundus autofluorescence (FAF) was performed after each laser session to assess the safety of panmacular micropulse laser treatment.

### 2.4. Statistical Analysis

Statistical analyses were performed using PQStat software (2021, PQStat v.1.8.2.208), while figures were generated using GraphPad Prism 8.4.3 (GraphPad Software, Inc., Sand Diego, CA, USA). Normality of data distribution was assessed us-ing the Shapiro–Wilk test (*p* < 0.05). Differences between sampling periods within the same object were assessed using the Wilcoxon matched pairs signed rank test (*p* < 0.05). In addition, the ANOVA Kruskal–Wallis test, followed by the Conover–Iman post hoc test, was used to determine the significance of the differences between objects within the same period. Spearman’s correlation coefficients were calculated to evaluate the relationship between the parameters evaluated.

## 3. Results

### 3.1. The Baseline Characteristic of the Groups

Finally, 41 patients with acute, recurrent, and chronic central serous chorioretinopathy (CSC) were included in the study. In addition, 14 healthy, age-matched individuals served as controls. Detailed demographic and clinical data of investigated groups of patients are presented in Table 1.

### 3.2. General Outcomes After Panmacular MPLT Treatment

After panmacular MPLT laser treatment, complete resolution of subretinal fluid (SRF) was observed in all aCSC patients (100%), 13 out of 15 cCSC patients (86.66%), and 7 out of 11 rCSC patients (63.63%).

In the aCSC group, the patients received an average of one laser treatment, ranging from one to three. Notably, nine aCSC patients required only a single laser treatment. On the other hand, the cCSC and rCSC groups had a higher average treatment burden, with an average of 2.8 treatments, ranging from one to eight. Eight cCSC, and six rCSC patients required only a single laser treatment. However, five and four cases, required more than three laser sessions in the cCSC and rCSC groups, respectively.

Despite treatment, two patients, one with rCSC and one with cCSC, never achieved even transient complete resolution of SRF. Moreover, one patient with cCSC and one with rCSC required eight laser treatments over two years, but complete fluid resolution was not achieved in these cases. In these refractory cases, patients reported persistent risk factors for CSC. In the case of the patient with rCSC, recurrence occurred during a period of significant personal stress, namely a divorce, while undergoing treatment for vitiligo of the skin. The patient with cCSC was engaged in shift work combined with physical exertion. It is important that both patients had CCT exceeding 450 µm and pachyvessel diameters exceeding 250 µm. However, it is reassuring that treatment maintained final BCVA at 1.0 and 0.8, and RetSen at 27.4 dB and 24.6 dB, respectively.

### 3.3. Changes in Structural and Microvascular Parameters in CSC Patients After MPLT Treatment

The study assessed functional, morphological, and vascular parameters both before treatment and two years after panmacular MPLT in patients with central serous chorioretinopathy (CSC), including acute (aCSC), chronic (cCSC), and recurrent (rCSC) cases.

#### 3.3.1. Functional Parameters

**Figure 3:**
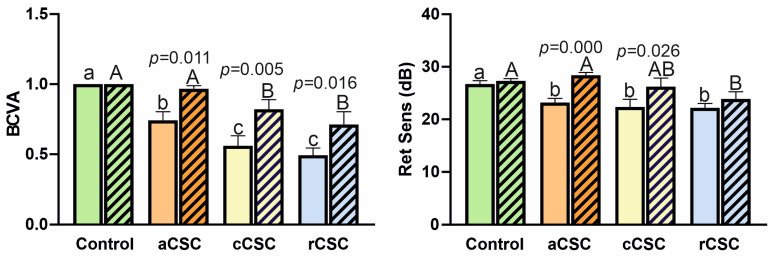
Comparison of best-corrected visual acuity (BCVA) and retinal sensitivity (RetSens) in patients with acute (aCSC), chronic (cCSC), and recurrent (rCSC) central serous chorioretinopathy at baseline and after two-year follow-up. Data are mean ± SE; values followed by different letters are significantly different (*p* < 0.05, Conover–Iman post hoc test). Lower case letters indicate differences between objects at the beginning of the observations, while upper case letters indicate differences between objects after 2 years. *p*-values are displayed for statistically significant differences between observation periods within each group, as assessed by the Wilcoxon matched-pairs signed-rank test.

Two functional parameters reflecting visual performance were assessed in healthy participants (controls) and in CSC patients: Best-Corrected Visual Acuity (BCVA) and Retinal Sensitivity (RetSens). The results are shown in Figure 3.

In the baseline analysis, there were significant differences in BCVA and RetSens between the control group and all CSC patients. Furthermore, BCVA was significantly lower in rCSC and cCSC compared to aCSC, while no statistically significant differences between CSC groups were observed in RetSens. After a 2-year follow-up, BCVA improvement was statistically significant in all treated patients, and RetSens was significantly improved in the aCSC (increase of 5 dB) and cCSC groups (increase of 3.8 dB). BCVA improved to levels comparable to healthy controls in aCSC, and RetSens in both aCSC and cCSC.

#### 3.3.2. Structural Parameters (Retinal and Choroidal)

**Figure 4:**
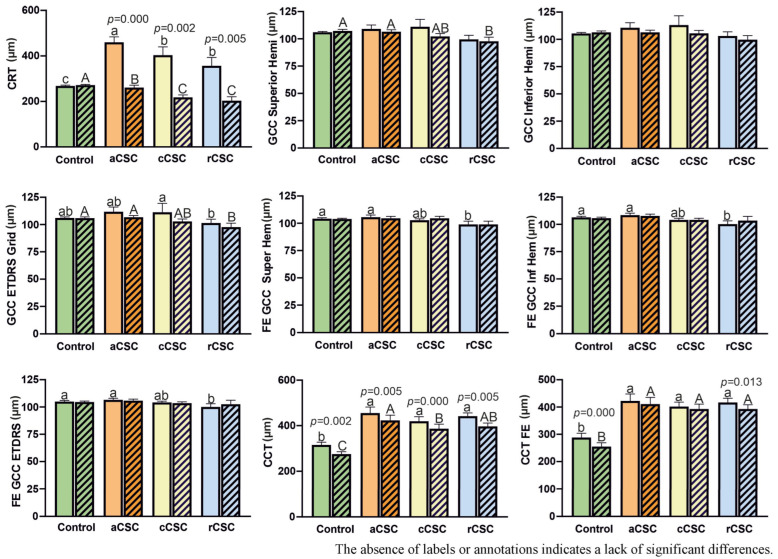
Comparison of structural parameters in patients with acute (aCSC), chronic (cCSC), and recurrent (rCSC) central serous chorioretinopathy at baseline two-year follow-up. Fe—fellow eye, CRT—Central Retinal Thickness, GCC—ganglion cell complex, Hemi-hemisphere, CCT—Central Choroidal Thickness. Data are mean ± SE; values followed by different letters are significantly different (*p* < 0.05, Conover–Iman post hoc test). Lower case letters indicate differences between objects at the beginning of the observations, while upper case letters indicate differences between objects after 2 years. *p*-values are displayed for statistically significant differences between observation periods within each group, as assessed by the Wilcoxon matched-pairs signed-rank test.

The variables analyzed included Central Retinal Thickness (CRT), Ganglion Cell Complex Thickness (GCC), Central Choroidal Thickness (CCT), and Pachyvessel Diameter. These parameters were analyzed to evaluate structural changes in the retina and choroid, which may reflect alterations in retinal integrity, ganglion cell loss, and choroidal vascular remodeling during follow-up. The results are shown in Figure 4.

At baseline, CRT was significantly higher in all CSC patient groups compared to the control group. Moreover, the mean CRT was significantly greater in the aCSC group than in the rCSC and cCSC groups. After two years, all patients showed a significant reduction in CRT compared to baseline. At the two-year follow-up, CRT in CSC patients was significantly thinner than in the control group.

At baseline, no statistically significant differences in GCC thickness (measured in the superior and inferior hemispheres) were observed between CSC and control groups. A similar trend was observed in the fellow eyes, with a slight reduction in GCC parameters limited to the rCSC group. No statistically significant changes in GCC measurements were found after the two-year follow-up.

At baseline, mean CCT was significantly greater in all CSC patients—both in affected and fellow eyes—compared to controls, with no significant differences among CSC subgroups. After two years, CCT significantly decreased in treated eyes, while changes in fellow eyes were minimal and statistically insignificant (except in the rCSC group). A slight decrease in CCT was also observed in the control group, likely related to aging [28].

At baseline, all CSC patients exhibited a significantly larger pachyvessel diameter than controls. In the fellow eyes, pachyvessel diameter was smaller than in eyes with active CSC; however, these differences among CSC subgroups did not reach statistical significance. After two years, a significant reduction in pachyvessel diameter was observed in the treated eyes of aCSC, rCSC, and cCSC patients, as well as in the fellow eyes of aCSC and rCSC patients.

#### 3.3.3. Retinal Microvascular Parameters

**Figure 5:**
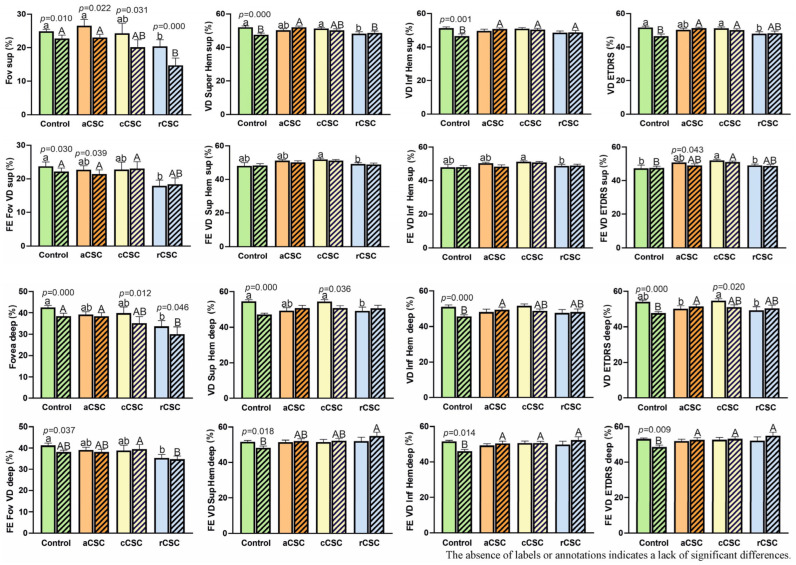
Comparison of vessel density (VD) of superficial plexus (sup) and deep plexus (deep) in patients with acute (aCSC), chronic (cCSC), and recurrent (rCSC) central serous chorioretinopathy at baseline two-year follow-up. Fe—fellow eye, Fov—fovea, Super Hem—superior hemisphere, Inf Hem—inferior hemi-sphere. Data are mean ± SE; values followed by different letters are significantly different (*p* < 0.05, Conover–Iman post hoc test). Lower case letters indicate differences between objects at the beginning of the observations, while upper case letters indicate differences between objects after 2 years. *p*-values are displayed for statistically significant differences between observation periods within each group, as assessed by the Wilcoxon matched-pairs signed-rank test.

The vascular parameters evaluated included superficial and deep foveal vessel density (SVD and DVD, respectively), vessel density in the superior and inferior hemispheres of both the superficial and deep capillary plexuses (VD Sup Hem, VD Inf Hem), and the Foveal Avascular Zone (FAZ). These parameters were analyzed to assess retinal perfusion and microvascular integrity. Comparison of results obtained for vessel density of the superficial and deep plexuses is demonstrated in Figure 5.

All groups had a similar baseline of superficial vascular parameters compared to the control group, in both the affected and fellow eyes, except for the rCSC group, which showed significantly lower values (Figure 5). After two years, a statistically significant de-crease in SVD was observed in the treated eyes of all patient groups, while in the fellow eyes, the changes were minor and reached statistical significance only in the control and aCSC groups.

Similar observation was for deep foveal vessel density. At baseline, DVD parameters showed no statistically significant differences between the control group and the aCSC or cCSC patients, in either the affected or fellow eyes, whereas the rCSC group again showed markedly lower values. After two years, a statistically significant decrease in DFVD was observed in the treated eyes of all patient groups. In the fellow eyes, the changes were minor and reached statistical significance only in the control group. These observations warrant further analysis. The decrease in vessel density may be related to age, but this change necessitates additional investigation.

In turn, FAZ analysis revealed no statistically significant variances between the groups for affected and fellow eye at the baseline or after the two-year follow-up. Furthermore, neither group observed any significant alterations over the two-year duration. However, at baseline and after two years, only the rCSC group had significantly larger FAZ compared to control for the affected and fellow eye (mean 0.3 ± 0.15 µm vs. 0.2 ± 0.04 µm, *p* = 0.046 and 0.36 ± 0.19 µm vs. 0.21 ± 0.06 µm, *p* = 0.011 for the affected eye, and 0.3 ± 0.1 µm vs. 0.21 ± 0.05 µm, *p* = 0.004 and 0.3 ± 0.1 µm vs. 0.21 ± 0.05 µm, *p* = 0.002 for the fellow eye).

### 3.4. Correlation Analysis

#### 3.4.1. Correlations Between Functional Parameters and Demographic and Clinical Data

Baseline and final Best-Corrected Visual Acuity (BCVA) showed significant negative correlations with Retinal Pigment Epithelium (RPE) alterations, Pigment Epithelium Detachment (PED), hyperreflective foci (HF), Ellipsoid Zone (EZ) disruptions, subretinal fluid (SRF) height, and patients’ smoking history (Table 2), while no significant correlations were observed with stress. These findings indicate that greater structural damage to the retina and the presence of morphological abnormalities are associated with poorer visual acuity, both before and after treatment. Retinal Sensitivity was negatively correlated only with smoking at baseline, suggesting that tobacco use may contribute to reduced retinal function.

#### 3.4.2. Correlations Between Functional, Vascular, and Morphological Parameters

The relationships between Retinal Sensitivity (RetSens), Best Corrected Visual Acuity (BCVA), and structural and vascular retinal parameters are summarized in Table 3.

RetSens showed significant positive correlations with CRT, superficial vessel density (VD) of the fovea, as well as the superior and inferior hemispheres (SupHem, InfHem) and ETDRS regions. After two years, RetSens remained positively associated with the entire deep plexus of the affected eye, the GCC of the affected eye, and the InfHem and ETDRS GCC of the fellow eye. Baseline RetSens also correlated positively with superficial and deep foveal VD and CRT at both time points, while negative correlations were observed with FAZ. Notably, final RetSens was positively linked to baseline GCC of the fellow eye (InfHem) and to superficial VD of the SupHem and ETDRS regions of the affected eye at two years.

Baseline BCVA correlated negatively with pachyvessel diameter, whereas final BCVA after two years showed positive correlations with GCC thickness of InfHem and ETDRS regions at both baseline and follow-up.

Overall, these findings indicate that better microvascular integrity, as reflected by vessel density in both superficial and deep layers—particularly in the foveal and ETDRS regions—is associated with improved visual function. Conversely, enlargement of the FAZ is linked to poorer BCVA and RetSens. Preservation of the Ganglion Cell Complex (GCC) appears crucial for visual recovery, as demonstrated by its positive correlations with both functional outcomes at the final visit.

## 4. Discussion

Central serous chorioretinopathy (CSC) is known to affect both structural and functional retinal parameters, which has a significant impact on visual performance and quality of life.

In our study, we aimed to evaluate the efficacy of panmacular MPLT in improving functional and structural retinal parameters in CSC patients. At the beginning of our investigation, significant differences were observed between healthy controls and CSC patients in Best-Corrected Visual Acuity (BCVA) and Retinal Sensitivity (RetSens). Both parameters were markedly reduced, indicating functional impairment associated with CSC. A similar deterioration in these parameters related to CSC has also been reported by other researchers [11,25,26,29,30]. Furthermore, pachyvessel diameter was significantly increased in CSC patients in both affected and fellow eyes, although it was significantly lower in the fellow eye.

Our study also revealed that panmacular MPLT 577 nm laser treatment was safe and effective for almost all CSC cases. Patients who received this treatment experienced significant and long-lasting improvements in their visual acuity across all studied group and average retinal sensitivity in most CSC cases, consistent with prior studies [10,25]. The average retinal sensitivity improved in aCSC and cCSC. Furthermore, the therapy reduced CCT and pachyvessel diameter. A similar decrease in CCT in aCSC treated with MPLT was reported earlier [10,30]. However, our study is the first to monitor pachyvessel diameter during MPLT treatment. Notably, the treatment was completely safe, as no damage to the RPE after the laser procedure was found at fundus autofluorescence (FAF), consistent with prior studies [12,13,25,26,30,31].

SRF was entirely resolved in all patients with aCS and most patients with cCSC and rCSC. Gawęcki et al. found similar results in acute CSC [25]. They ultimately reduced SRF in 81.25% of CSC cases, lasting an average of 3.4 ± 2.3 months. However, laser therapy was performed according to the SCOT map under the edoema. Similarly, Kiraly et al. limited laser therapy to the area of the neurosensory retinal detachment. However, in these cases, total SRF resolution was achieved only in 48.4% of CSC cases lasting three months [26]. The treatment significantly improved mean BCVA, especially in patients with a complete reduction in SRF, during the six-month follow-up period [25,26]. After a two-year follow-up, we also found that the treatment was the most effective for patients suffering from acute CSC. In our assessment, the panmacular application of the MPLT laser seems to harness MPLT’s known effects more comprehensively, particularly in stimulating RPE and Müller cells. In our study, among the CSC groups, those with acute CSC demonstrated complete SRF resorption and the most significant enhancement, achieving BCVA levels and RetSens comparable to control at the two-year follow-up. For them and cCSC patients, the change in BCVA was the most significant.

Conversely, patients with rCSC exhibited the slightest improvement in BCVA and RetSens. However, this group initially presented the worst visual acuity and retinal sensitivity. Moreover, their baseline pachyvessel diameter was the largest, persisting even after the two-year follow-up. Interestingly, while CCT was greatest in patients with aCSC at baseline, the rCSC group closely followed, displaying a CCT larger than that of the non-treated and cCSC groups. Although the change in mean CCT in rCSC patients was most remarkable after treatment, their CCT was still greater than that of the cCSC and non-treated CSC groups after two years.

After treatment, all CSC groups showed significant reductions in pachyvessel diameter. Retinal sensitivity also improved in all treated and untreated CSC patients after two years. However, the most significant improvement was observed in patients of the aCSC group, followed by cCSC. Only the rCSC group showed a marginal improvement. Moreover, those patients had the lowest RetSens at baseline and after two years of follow-up but without statistical significance. Li et al. observed similar but significant differences in retinal sensitivity between rCSC and aCSC [29]. Similarly, Go et al. reported that the improvement in retinal light sensitivity was greater in treated aCSC patients than in untreated patients [30].

The final BCVA depended on the GCC’s thickness measured at baseline and after two years for the inferior hemisphere and ETDRS Grid. The mean GCC thickness of the ETDRS Grid was lowest in the rCSC group at baseline and after two years.

Moreover, baseline BCVA was negatively correlated with pachyvessel diameter, the largest in the rCSC group before and after panmacular MPLT treatment. However, the difference between the groups was insignificant for the affected and fellow eyes, either at baseline or after two years.

Notably, a significant difference was noted between cCSC and rCSC groups in the superior hemisphere of the superficial plexus, pointing to a weaker vascular network in rCSC. A decrease in the density of the superficial and deep vascular plexuses in the fovea was observed in both the control group and treated patients with CSC at similar levels, suggesting a common aging effect associated with beneficial treatment-related changes [32], except in the rCSC group, which showed a greater decrease in VD of the superficial plexus. This finding highlights the more invasive course of the disease in that group. On the other hand, in the treated aCSC group, the decrease in the VD of the deep plexus in the fovea was less pronounced than in the control group, suggesting a beneficial effect of both shorter disease duration and treatment received.

Patients with recurrent CSC exhibited the lowest initial and final BCVA and RetSens, prompting the question: Why is this the case? Traditionally, it was thought that the duration of the disease process was the primary risk factor, leading to damage to RPE cells and photoreceptors. Indeed, our analysis has shown a clear negative correlation between the initial or final BCVA and disease duration. We found that the disease duration tends to be longer in patients with rCSC than cCSC, which may seem unexpected. It is important to note that previous studies often grouped rCSC patients with the cCSC group, which could have distorted the results.

The poorer outcomes observed in rCSC patients compared to cCSC may be attributed to the widest pachyvessels in these patients. It is established that atrophy and thinning of the choriocapillaris occur over large choroidal vessels, leading to RPE damage and SRF accumulation [33]. Compression of the choriocapillaris may cause a reduction in vascular flow area, while Sattler’s and Haller’s layers reveal increased blood flow [33].

After analyzing the data, we recommend prompt treatment for rCSC patients. In the two-year follow-up, the disease’s progression appears to be considerably more severe in such cases. We strongly concur with Gawęcki et al. that timely intervention in all CSC cases may help prevent permanent visual acuity reduction and lead to beneficial outcomes [25].

Among the parameters examined, RPE alterations and EZ disruptions were most commonly noted in the cCSC group despite exhibiting superior BCVA and RetSens compared to rCSC at baseline and after two years. Notably, when analyzing disease duration from the initial known episode in the rCSC group, this parameter was more prolonged than in the cCSC group. The presence of RPE alteration is still closely related to the definition of the chronic form of CSC, in which these changes may be more extensive, including retinal atrophy. However, in our study, cCSC patients had better baseline BCVA and RetSens than rCSC patients. Moreover, despite the chronicity of the process, they achieved significantly better RetSens, which we did not observe in the rCSC group. Additionally, in rCSC group the improvement in RetSens was not statistically significant.

Thus, the disease duration appears to exert the most significant influence on the final BCVA. These observations align with findings from other studies [12,25]. Therefore, minimizing disease duration is crucial, primarily through simple and safe methods like subthreshold MPLT.

In our opinion, subthreshold panmacular MPLT is the most straightforward, safest, and least invasive choice of the current therapeutic options. The safety was confirmed by our and prior studies [13,19,21].

There are some limitations of the study. The number of participants in the study group was relatively small. Further studies with more participants and consistent treatment regimens should be conducted to confirm these findings. Although a two-year follow-up period provides valuable insights, extending it may supply additional information regarding the course of the disease, the recurrence rate, and the need for further treatment. Another limitation is the observed statistically significant decrease in vessel density (VD) in the superficial and deep plexuses across all groups, including the control group. Explaining this phenomenon as an ‘effect of aging’ seems insufficient, given the short 2-year follow-up period. A more probable explanation involves measurement artifacts related to OCT-A technology. The presence of subretinal fluid (SRF) at the baseline measurement could have caused segmentation errors in the automated detection of vascular plexuses, leading to artificially inflated VD values. After SRF resolution, the final measurement, performed on a retina with correct anatomy, was more accurate but consequently appeared lower. Therefore, the observed changes in VD must be interpreted with great caution, as they may reflect the limitations of the measurement technology rather than an actual physiological phenomenon analysis was abandoned. Consequently, all findings related to the decrease in VD over time (e.g., in. Figure 5), and all correlation based on these changing values (e.g., Table 3), must be interpreted with extreme caution and are likely confounded by this measurement artifact. The true effect of MPLT on retinal microvasculature remains inconclusive based on this data.

## 5. Conclusions

Panmacular MPLT treatment appears to induce long-term improvement of morphological parameters (reduction in CCT and pachyvessel diameter) and functional parameters (improvement in BCVA) in patients with active CSC. A key finding of this study is the significant difference in outcomes based on disease subtype. Patients with recurrent CSC (rCSC) exhibited the poorest baseline function and weakest therapeutic response, with improvements in retinal sensitivity (RetSens) not reaching statistical significance. This suggests that a recurrent disease course, possibly linked to more severe baseline choroidal abnormalities, may be a primary negative prognostic factor. Conversely, the greatest functional improvement was demonstrated in aCSC patients. However, due to methodological limitations (the lack of a randomized control group), this demonstrates efficacy in patients who fail to resolve spontaneously, rather than superiority over a longer observation-only period. Future randomized controlled trials comparing early intervention strategies in aCSC with conservative observation are necessary to establish the gold standard of care. The procedure is safe and could be considered an economically preferred treatment option for active CSC cases. Timely treatment appears crucial for beneficial outcomes, especially in preventing the progression to recurrent or chronic forms which demonstrate poorer functional recovery.

## Figures and Tables

**Table 1:** Baseline demographics and clinical data of central serous chorioretinopathy (CSC) patients and healthy controls. CSC subtypes: acute (aCSC), chronic (cCSC), and recurrent (rCSC).

Parameter	Control	aCSC	cCSC	rCSC	*p*
Number of patients (n, %)	14 (25.5)	15 (27.3)	15 (27.3)	11 (20.0)	—
Age (years ± SD)	45 ± 9.26	40.9 ± 5.63	46.8 ± 6.58	44 ± 8.96	0.264
Sex, male (n, %)	8 (57.14)	13 (86.6)	8 (53.33)	7 (63.33)	0.988
Residence area, urban (n, %)	8 (57.14)	6 (40.0)	11 (73.33)	7 (63.63)	0.460
Duration of symptoms (months ± SD)	0	1.6 ± 0.90	15.64 ± 20.15	17.45 ± 25.47	0.000
Stress, yes (n, %)	4 (28.57)	2 (13.33)	2 (13.33)	4 (36.36)	0.190
Smoking history, yes (n, %)	0	2 (13.33)	4 (26.66)	3 (27.27)	0.110
RPE alteration, yes (n, %)	0	2 (13.33)	12 (80.0)	10 (90.91)	0.000
PED, yes (n, %)	0	2 (13.33)	3 (20.0)	1 (9.09)	0.480
HF, yes (n, %)	0	11 (73.33)	14 (93.33)	10 (90.91)	0.000
EZ disruptions, yes (n, %)	0	3 (20.0)	5 (33.33)	3 (27.27)	0.240

RPE—Retinal Pigment Epithelium; PED—Pigment Epithelium Detachment; HF—Hyperreflective Foci; EZ—Ellipsoid Zone.

**Table 2:** Spearman correlation between Best Corrected Visual Acuity (BCVA), Retinal Sensitivity (RetSens), and patient demographic and clinical data (R Spearman, *p* < 0.05 *).

	BCVA		RetSens	
Variables	Baseline	Final	Baseline	Final
Age	−0.198 *p* = 0.113	−0.337 * *p* = 0.006	0.031 *p* = 0.874	−0.199 *p* = 0.159
Disease duration/months	−0.420 * *p* = 0.002	−0.447 * *p* = 0.000	−0.091 *p* = 0.522	−0.263 *p* = 0.062
RPE alteration, yes	−0.614 * *p* = 0.000	−0.498 * *p* = 0.000	−0.099 *p* = 0.474	−0.261 *p* = 0.63
PED, yes	−0.347 * *p* = 0.005	−0.293 * *p* = 0.017	−0.040 *p* = 0.701	−0.344 * *p* = 0.013
HF, yes	−0.596 * *p* = 0.000	−0.548 * *p* = 0.000	−0.215 *p* = 0.130	−0.123 *p* = 0.386
EZ disruptions, yes	−0.525 * *p* = 0.000	−0.402 * *p* = 0.000	−0.030 *p* = 0.781	−0.058 *p* = 0.685
SRF height	−0.394 * *p* = 0.001	−0.267 * *p* = 0.031	−0.108 *p* = 0.590	0.003 *p* = 0.982
Stress, yes	−0.020 *p* = 0.875	−0.159 *p* = 0.210	−0.013 *p* = 0.920	−0.150 *p* = 0.236
Smoking history	−0.354 * *p* = 0.004	−0.320 * *p* = 0.010	−0.339 * *p* = 0.006	−0.194 *p* = 0.124

RPE—Retinal Pigment Epithelium; PED—Pigment Epithelium Detachment; HF—Hyperreflective Foci; EZ—Ellipsoid Zone; SRF—Subretinal Fluid.

**Table 3:** Spearman correlation between Best Corrected Visual Acuity (BCVA), Retinal Sensitivity (RetSens), and vascular and morphological parameters (R Spearman, *p* < 0.05 *).

	BCVA		RetSens	
Variables	Baseline Baseline/Final	Final Baseline/Final	Baseline Baseline/Final	Final Baseline/Final
CRT	−0.127/0.595 *	0.031/0.371 *	−0.043/0.233	0.285 */0.447 *
*p*-value	0.313/0.000	0.801/0.002	0.759/0.99	0.042/0.000
FAZ	−0.277 */−0.300 *	−0.273 */−0.178	0.089/0.053	−0.334 */−0.369 *
*p*-value	0.025/0.015	0.027/0.155	0.533/0.711	0.016/0.008
Superficial VD Fovea	0.393 */0.458 *	0.380 */0.434 *	−0.122/−0.009	0.286 */0.298 *
*p*-value	0.001/0.000	0.001/0.000	0.393/0.533	0.041/0.033
Superficial VD SupHem	0.243/−0.015	0.382 */0.271 *	0.031/0.230	0.165/0.412 *
*p*-value	0.051/0.903	0.001/0.029	0.827/0.104	0.246/0.003
Superficial VD InfHem	0.252 */−0.011	0.398 */0.213	−0.050/0.137	0.083/0.258
*p*-value	0.042/0.903	0.001/0.104	0.724/0.335	0.558/0.067
Superficial VD ETDRS	0.234/−0.004	0.381 */0.277 *	−0.017/0.180	0.136/0.383 *
*p*-value	0.059/0.972	0.001/0.025	0.903/0.204	0.341/0.005
Deep VD Fovea	0.375 */0.398 *	0.477 */0.464 *	−0.112/0.098	0.383 */0.537 *
*p*-value	0.002/0.001	0.000/0.000	0.430/0.490	0.005/0.000
Deep VD SupHem	0.092/−0.023	0.332 */0.257 *	−0.073/0.050	0.033/0.128
*p*-value	0.462/0.851	0.006/0.038	0.606/0.724	0.814/0.368
Deep VD InfHem	0.122/0.032	0.335 */0.260 *	−0.085/0.051	0.027/0.128
*p*-value	0.329/0.799	0.006/0.038	0.551/0.724	0.849/0.369
Deep VD ETDRS	0.122/0.071	0.361 */0.344 *	−0.078/0.070	0.034/0.188
*p*-value	0.329/0.571	0.003/0.004	0.578/0.624	0.809/0.186
Pachyvessel diameter	−0.341 */−0.357 *	−0.086/−0.110	−0.183/−0.246	−0.147/−0.197
*p*-value	0.005/0.003	0.494/0.380	0.197/0.081	0.302/0.164
GCC SupHem	0.063/0.206	0.214/0.242	0.208/0.352 *	0.359 */0.425 *
*p*-value	0.613/0.098	0.086/0.051	0.141/0.11	0.009/0.001
GCC InfHem	0.144/0.244	0.283 */0.268 *	0.226/0.530 *	0.359 */0.546 *
*p*-value	0.249/0.051	0.022/0.031	0.110/0.000	0.009/0.000
GCC ETDRS	0.111/0.197	0.286 */0.303 *	0.226/0.437 *	0.369 */0.494 *
*p*-value	0.376/0.114	0.021/0.014	0.110/0.001	0.007/0.000

CRT—central retinal thickness; FAZ—foveal avascular zone; VD—vascular density; SupHem—superior hemisphere; InfHem—inferior hemisphere; GCC—ganglion cell complex thickness; ETDRS—grid for retinal assessment.

## Data Availability

The data presented in this study are available on request from the corresponding author.
